# Quantifying the Extent of Calcification of a Coccolithophore Using a Coulter Counter

**DOI:** 10.1021/acs.analchem.2c01971

**Published:** 2022-09-08

**Authors:** Xinmeng Fan, Christopher Batchelor-McAuley, Minjun Yang, Samuel Barton, Rosalind E. M. Rickaby, Heather A. Bouman, Richard G. Compton

**Affiliations:** † Physical and Theoretical Chemistry Laboratory, Department of Chemistry, University of Oxford, South Parks Road, Oxford OX1 3QZ, Great Britain; ‡ Department of Earth Sciences, University of Oxford, South Parks Road, Oxford OX1 3AN, Great Britain

## Abstract

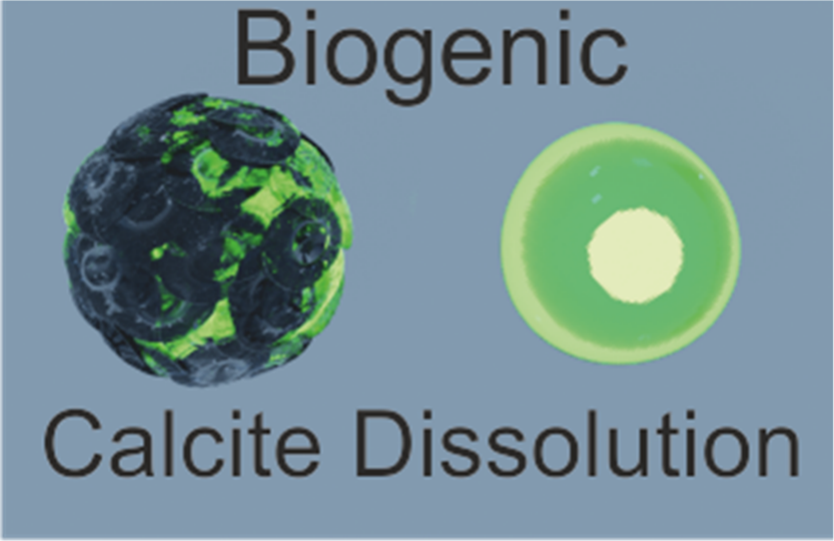

Although, in principle, the Coulter Counter technique yields an absolute measure of particle volume, in practice, calibration is near-universally employed. For regularly shaped and non-biological samples, the use of latex beads for calibration can provide sufficient accuracy. However, this is not the case with particles encased in biogenically formed calcite. To date, there has been no effective route by which a Coulter Counter can be calibrated to enable the calcification of coccolithophores—single cells encrusted with biogenic calcite—to be quantified. Consequently, herein, we seek to answer the following question: *to what extent can a Coulter Counter be used to provide accurate information regarding the calcite content of a single*-*species coccolithophore population?* Through the development of a new calibration methodology, based on the measurement and dynamic tracking of the acid-driven calcite dissolution reaction, a route by which the cellular calcite content can be determined is presented. This new method allows, for the first time, a Coulter Counter to be used to yield an absolute measurement of the amount of calcite per cell.

## Introduction

**Figure 1:**
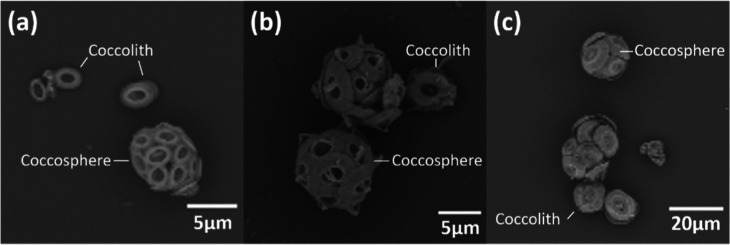
Figure showing SEM images of three coccolithophore species and their detached coccoliths (a) *E. huxleyi,* (b) *G. oceanica,* and (c) *C. braarudii*.

Coulter Counters and related resistive-pulse sensing devices are routinely used in a range of different fields that require the counting and sizing of particles, microbes, and molecules.^1^ These devices use the changes in an ionic current through an orifice in an electrolyte medium, caused by a traversing particle, to yield information about the number and size of the particles in a sample. The use of this technique principally stems from the rapidity and accuracy with which it can provide particle counts. In the oceanic science community, these devices are often employed during the growth of phytoplankton cell cultures to provide a cell count capable of tracking the growth of the cell population.^2,3^ Although it has long been recognized that such Coulter Counters can provide quantitative information about the particle dimensions,^4^ when it comes to irregularly shaped materials, precisely and accurately relating the measured “equivalent spherical diameter” directly to the mass or volume of the material is not straightforward.^5^ Specifically, in terms of ocean science, the amount of calcite produced by individual coccolithophores is an important parameter for the biogeochemical cycle of carbon but is an experimentally challenging measurement. At the single cellular level, biogenically formed calcite is extruded from the phytoplankton as coccoliths (liths),^6^ nanostructured plates, onto the outer surface of the cell (see Figure 1 for SEM images of calcified coccolithophores and detached coccoliths). This calcite is thought to provide protection for the cell, and en masse, these planktons globally produce calcite of the order of 10^15^ g per year and very significantly contribute to the marine carbon cycle and global CO_2_ fixation fluxes.^7^

In the laboratory environment, methods have been developed to quantify this biogenic calcite of individual cells including the use of optical polarization,^8^ X-ray tomography,^9^ filtering,^10^ and ICPOES^11^ (inductively coupled plasma optical emission spectroscopy). However, none of these technologies lend themselves to use in routine analysis of the biogenic calcite mass of coccolithophores, either limited by the method itself^12^ or requiring specialized equipment and/or analysis methods. Here, we seek to use an alternative approach and explore *to what extent a Coulter Counter can be used to provide accurate information regarding the calcite content of a coccolithophore population.*

**Figure 2:**
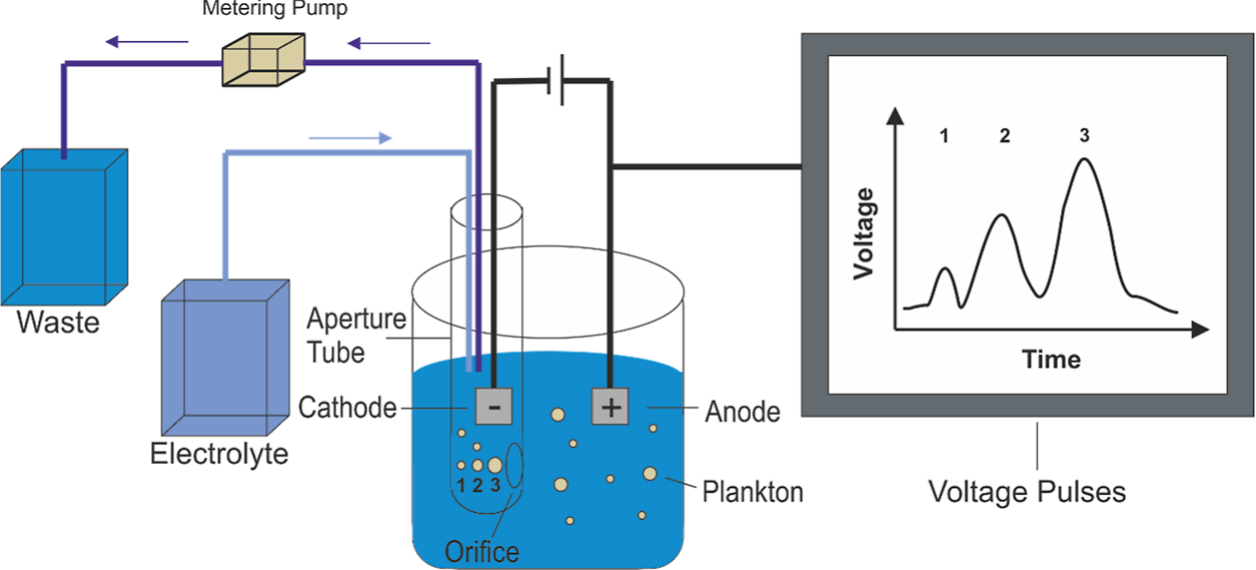
Schematic diagram of the Coulter Counter experiment showing how the plankton are in a suspension and are then pumped through an orifice. The change in the electrical resistance of the orifice leads to pulses in the voltage time profile as measured using the Coulter Counter. For a given particle shape, the magnitude of the pulse is proportional to the volume of the particle traversing the orifice.

As shown schematically in Figure 2, a Coulter Counter consists of two separate chambers filled with an ionically conductive solution, and the chambers are connected via an orifice. In each chamber, there is a platinum foil electrode which creates an electrochemical cell. A potential is applied across the two electrodes, inducing an ionic current to flow. The orifice essentially acts as an ionic resistor where in most experimental setups the Coulter Counter applies a fixed ionic current through the orifice and modulates the potential applied across the cell accordingly. The potential required to pass a given current is proportional to the conductivity of the electrolyte but also importantly depends upon the size and geometry of the used orifice. In a Coulter Counter experiment, one of the chambers contains a particle suspension, and a pump is used to pass a controlled volume of the particle suspension through the orifice. A particle passing through the orifice leads to a measurable change or “pulse” in the ionic resistance which is recorded using the device. First, counting the number of pulse events gives a direct measure of the number of particles in a given volume (concentration). Second, the pulse height itself can yield information about the *volume* of the particle passing through the orifice. In principle, the magnitude of the pulse is absolute, and analytical solutions for simple particle geometries have been provided, where the pulse height is proportional to the particle volume multiplied by some shape factor.^4,13^ However, complications arise even in the simple case of oblate spheroids where the magnitude of the pulse varies as a function of the orientation of the particle relative to the orifice.^14^ Furthermore, as will be most relevant for this work, in the case of porous materials, the pulse magnitude often more closely reflects not the volume of the solid but the entire envelope volume; the electrolyte is occluded in the porous structure and does not significantly contribute to the conductivity of the orifice.^15,16^ Placing the nuances of the fundamental aspects of the Coulter Counter measurement aside, although the measurement technique is absolute, in practice, device calibration is near-universally employed.^5^ This calibration is used to provide correspondence between the pulse magnitudes and the particle volumes. There are two principal calibration procedures: first, the use of latex microspheres of well-defined shape and size and second—as more often advocated in the older literature—the mass integration method. The first method relates the measured pulse height to that expected for an ideal spherical particle, and the reported dimensions are those of the equivalent sphere. Hence, the measured particle dimension is only accurate if the particle is itself non-deformable, solid, and spherical. The second method involves taking a sample of known density, suspending a known mass in a given volume, measuring the sample with a Coulter Counter, and relating the sum of the measured volume (from the Coulter Counter) to that of the known values for the sample.^17^ Although more accurate, especially for non-spherical or porous materials, this calibration is specific to a given material and sample.

The following question arises therefore: for calcified coccolithophores such as those presented in Figure 1, what does the Coulter Counter measure and can a route be found to calibrate this measurement? Note, as highlighted in Supporting Information Section S1, that there seems to be a range of views in the recent literature as to what can and cannot be measured using a Coulter Counter, where in one extreme case in the literature, it was claimed that the calcite shell is not experimentally observable using this technique.^18^ In the present work, we first unambiguously demonstrate that the Coulter Counter does in fact measure the calcite on a calcified coccolithophore and then second provide a new route by which this measurement can be calibrated yielding an absolute, as opposed to relative, measure of the calcite per cell. This new technique is based on experimentally monitoring the in situ acid-driven calcite dissolution reaction. Recent work has demonstrated how, by optically monitoring the acid-driven dissolution of the calcite on individual coccolithophores, the mass of calcite can be directly inferred from the kinetics of the dissolution process.^12^ In this work, we use this acid dissolution process and monitor it using a Coulter Counter; this new methodology provides a direct route by which the calcite content per cell can be accurately and routinely measured.

## Experimental Section

### Materials

Three species of coccolithophores were obtained from the Roscoff Culture Collection (RCC, France): *Emiliania huxleyi* (RCC1216), *Gephyrocapsa oceanica* (RCC1314), and *Coccolithus braarudii* (RCC1198). The details of the culturing conditions are provided in Supporting Information Section S2.

For all solutions, Milli-Q ultrapure water with a resistivity of 18.2 MΩ cm at 25 °C was used. NaCl was purchased from Fisher Bioreagents, NaHCO_3_ from Acros Organics, and CaCl_2_ from Aldrich. TRIS [(HOCH_2_)_3_CNH_2_], acetic acid, and sodium acetate came from Aldrich. All solutions were filtered using 0.45 μm filters and adjusted to pH 8 with HCl (Fisher Chemical) and NaOH (Honeywell).

### Coulter Counter Measurements

A Multisizer 4 Particle Analyzer (PN A51387A, Beckman Coulter, Inc., U.S.) was used for the Coulter Counter measurements. An 800 μA current was applied using the Coulter Counter. Static and dynamic assessment (see the Results and Discussion section for more details) of the sample was done in small and large volumes, respectively. For a small volume, a 0.5 mL sample was added to 20 mL of the electrolyte. For a large volume, a 1.5 mL sample was injected into 100 mL of the electrolyte with a stirrer.

### Optical Microscopy Measurements

Optical measurements were made on an Axio Examiner A1 microscope (Carl Zeiss Ltd., Cambridge, U.K.). A 40× oil immersion objective (Plan-Apochromat 40×/1.3 Oil Iris) was used for *E. huxleyi* and *G. oceanica* samples, and a 20× objective (Plan-Apochromat 20×/0.75) was used for *C. braarudii* samples. The brightfield illumination was applied, and an ORCA-Flash 4.0 digital CMOS camera (Hamamatsu, Japan) was used for the image acquisition.

The images were analyzed using ImageJ freeware (Fiji). The projection area of each coccolithophore in pixels is determined by thresholding the edge manually. The actual projection area is the number of pixels in the 2-D image multiplied by the pixel resolution (0.155 × 0.155 μm^2^ pixel^–1^ for the 40× objective and 0.315 × 0.315 μm^2^ pixel^–1^ for the 20x objective).

### Data Analysis

To calculate the shape factor for each species on a typical day, first, an assessment of the size of the coccolithophores with and without their shells was undertaken using the Coulter Counter. The Coulter Counter reports the particle size distribution as measured from the transient pulse heights caused by the particles traversing through the sensing orifice. From these cell size distribution plots, it was possible to determine the mean and standard deviations (σ) of the cell sizes as directly measured using the Coulter Counter. Having assessed the range of cell sizes present in a sample, a kinetic measurement of the shell dissolution (dynamic assessment, see below) was made to determine the dissolution time under acetic acid conditions. Here, the coccolithophore cells were injected into the electrolyte while the Coulter Counter was measuring the solution-phase particle size. For each run, 20,000 pulses were obtained as a function of time. These individual pulses were subsequently filtered to remove outliers and data points that likely do not correspond to coccolithophore cells. These nonplankton-related pulses arise due to background noise, air bubbles, and detritus from the plankton growth in the solution. This electronic filtering was done on the basis of the previously measured means and standard deviations of the cells as obtained from the earlier static measurement. Any of the pulses that are larger than the mean size of the coccolithophore with a shell plus 3σ or smaller than the mean size of the deshelled coccolithophore minus 3σ were removed from the data set. After filtering, there were around 50–150 data points/second. The windowed average of this data set was calculated from the filtered data set where a window size of 50 points was used and then plotted as a function of time. The dissolution time is extracted from this plot. Third, the optical radius with and without shells was measured on the same day. Based on the measured optical radius and dissolution time from the dynamic assessment, the expected volume of the calcite shell can be calculated. As discussed later in the text, the shape factor is equal to expected shell volume from dynamic measurement of the calcite volume divided by that from static assessment, giving a measure of the extent by which the Coulter Counter overestimates the amount of calcite per cell.

## Results and Discussion

In the following sections, first, we evidence that the Coulter Counter is sensitive to the presence of calcite on a coccolithophore and report the calcite volume, and hence mass per cell, as directly measured via the Coulter Counter. Doing so also requires a brief discussion of the importance of the used electrolyte. Herein, we refer to this conventional Coulter Counter measurement as the “static assessment” of the calcite mass, where the calcite mass is inferred from the difference in the magnitude of the Coulter Counter pulses before and after dissolution of the coccolithophore shell. The term static is principally used to reflect the fact that the calcite mass per cell does not change during the course of the measurement, and the cell is either fully shelled or deshelled in the Coulter Counter measurement. Having done this, it is next demonstrated how a Coulter Counter can be employed to make kinetic measurements of the calcite shell dissolving in a weak acid solution and thus allow the relative size of a coccolithophore population to be tracked as the calcite shell is dissolved. This new technique is referred to as the “dynamic assessment” of the coccolithophore calcite content, where the term dynamic is used to reflect the fact that the cellular calcite content changes during the course of the measurement. Here, the total calcite mass is inferred from the time required for the coccolithophore shell to dissolve; this dissolution process is monitored via the change in the magnitude of the Coulter Counter pulse sizes. It should be emphasized that in this dynamic assessment, the magnitude of the Coulter Counter pulse is not used to *directly* infer the size of the material traversing the orifice but is simply used as a method by which the end point of the reaction can be ascertained. Finally, using this dynamic Coulter Counter technique, three coccolithophore species are tracked during the incubation of the sample demonstrating the applicability of the new dynamic measurement of coccolithophores of massively differing sizes. This further allows us to consider the proportionality between the static Coulter Counter-measured calcite volume and the actual volume of the CaCO_3_ material as reported by the newly developed dynamic measurement process.

### Static Assessment of the Coccolithophore Calcite Content

**Figure 3:**
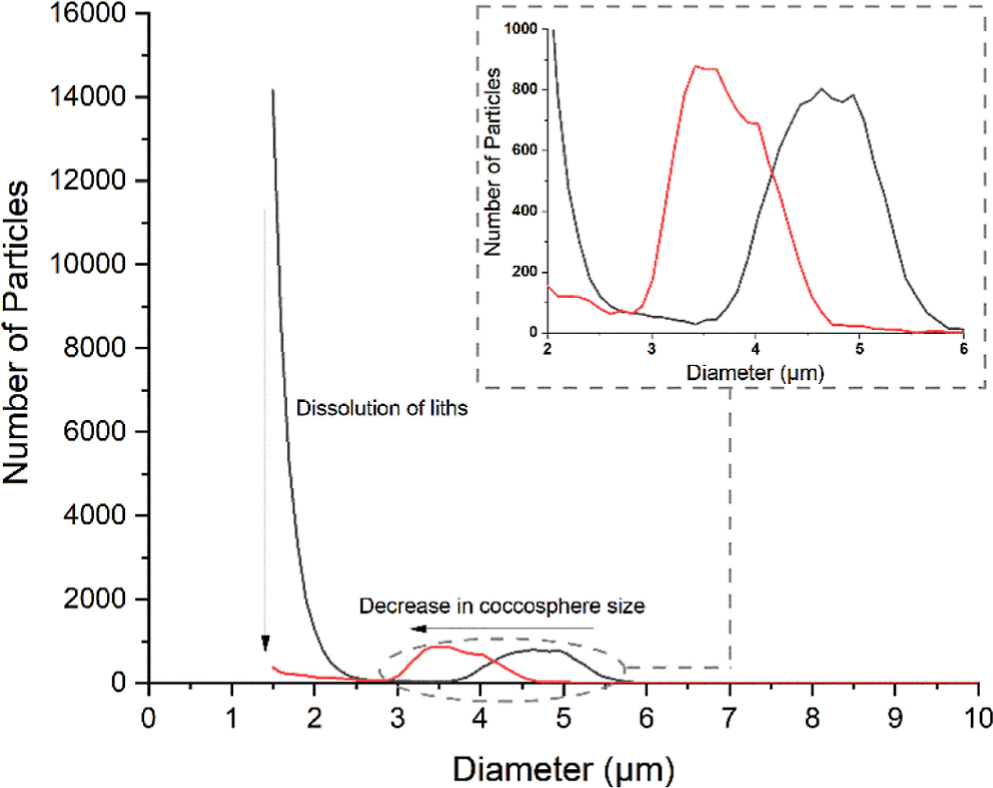
Measured size distribution for *E. huxleyi* in the presence (red line) and absence (black line) of the addition of 10 mM HCl to the electrolyte as directly measured using the Coulter Counter. The size distribution is obtained using the Coulter Counter by recording the size of the individual pulses associated with particles traversing across the measurement orifice, and the resulting voltage pulses are converted to an effective particle diameter using a predefined conversion factor obtained from calibration of the device with latex spheres. Herein, we refer to this measurement as a “static” measurement of the particle size distribution as the size of the particles does not change during the course of the experiment.

The choice of the electrolyte for a Coulter Counter measurement can be an important factor under some circumstances; this is true to the extent that the international standard provides an extensive list of suggested electrolytes for use with different materials.^17^ The ISOTON II diluent is a regularly used electrolyte suitable for counting red blood cells; however, this commercial product uses a phosphate buffer which in the present case is unsuitable due to the presence of calcium ions in the coccolithophore sample solution, which would lead to the precipitation of calcium phosphate. The Coulter Counter technique is intrinsically an electrochemically driven measurement where conventionally two large platinum foils are used to apply a potential across the orifice. Consequently, the presence of a buffer in the electrolyte solution is important for longer-duration experiments (see Supporting Information Section S3 for further details); during the course of the measurement, a significant concentration of protons can be formed at the anode by oxidation of water. Furthermore, in the present work, we wish to quantify the amount of calcite present in the suspension, so it is imperative to ensure that the solution is at least saturated with respect to calcite, otherwise undersaturation of the solution will lead to dissolution of the material. In the following work, we use an electrolyte of 4% NaCl, 10.0 mM Tris buffer, 20.0 mM CaCl_2_, and 1.0 mM NaHCO_3_, where the electrolyte has been both filtered and adjusted to pH 8.0. NaCl is required to ensure that the solution is suitably conductive for the Coulter Counter. The Tris buffer is used to ensure that the pH of the solution is maintained at pH 8.0 during the course of the experiment, and the calcium and bicarbonate are used to ensure that the calcite does not dissolve due to undersaturation of the solution. Using this electrolyte, the size distribution of an *E. huxleyi* sample was measured using the Coulter Counter, where the diameter is reported relative to that of an equivalent solid sphere. This measurement was repeated with the addition of 10.0 mM HCl to the solution, the results of which are shown in Figure 3. Here, the particle size distribution is reported using the Coulter Counter and is attained through measurement of the individual pulse heights and conversion of these voltage pulses to effective particle diameters. The proportionality between the pulse size and the effective particle diameter is provided though calibration of the device with latex spheres. Herein, we refer to this direct measurement of the size of the particle in solution as a “static assessment” of the particle size. Specifically, by referring to the measurement as static, we are highlighting the fact that in contrast to the dynamic measurement developed below, the particle size does not change during the course of the measurement.

In the pH 8.0 calcite-saturated solution, the size distribution exhibits two main features. First, at a relative diameter of 4.7 ± 0.5 μm, there is a broad peak associated with the individual coccolithophores. Second, at around 1 μm, close to the limit of resolution with a 70 μm diameter orifice, there is a high particle count where this feature reflects the presence of detached liths in the solution phase. The addition of 10 mM HCl to the solution causes the buffer to be overwhelmed and the measured pH of the solution to drop to ∼2. As can be seen in Figure 3, the addition of the acid decreases the size of the main peak to a relative diameter of 3.7 ± 0.4 μm, and the feature associated with individual liths at the lower end of the size distribution is completely removed. This change in the position of the main peak and the removal of the individual free liths arise due to dissolution of the biogenic calcite by the strong acid. The removal of the coccolithophore shell leads to a measurable decrease in the cell volume.

It is beneficial to compare these measured relative diameters to those obtained from optical microscopy as summarized in Table 1 (see the Experimental Section for details on these measurements). In both cases, with or without the addition of the acid, the relative diameters as measured using the Coulter Counter are 20–30% lower than those measured optically. This propensity for the Coulter Counter to underestimate phytoplankton cell diameters has been noted previously (see Supporting Information Section S1). Given that the Coulter measurement is proportional to the particle volume,^4^ this represents over a factor of 2 error in the reported volume. Ultimately, this discrepancy reflects the limitations of using latex microspheres to calibrate the measurement. There are a number of different factors that contribute to why such biological particles are not well modeled as ideal microspheres. A primary issue is the relative deformability of biological cells.^4,19^ In the present work, a solution flow rate of 18–20 μL s^–1^ has been used, given that the orifice has a diameter of 70 μm; this implies a solution velocity in the sensing region of the order of 5 m s^–1^. Such high flow rates are known to lead to significant distortion of mammalian cells in Coulter Counters leading to significant underestimation of the cell volume.^4,19^ Clearly, when the Coulter Counter has been calibrated using latex microspheres, the measured cell volumes and hence diameter of the biological cells are not accurate, and the reported changes in size are relative as opposed to absolute. The following question then arises: given that the Coulter Counter measurement is sensitive to the presence of the calcite shell, is it possible to use this technique to attain an accurate measurement of the cellular calcite content?

**Table 1:** Measured Diameters for *E. huxleyi* Obtained from the Coulter Counter and Optical Microscopy

	before addition of 10 mM HCl (μm)	after addition of 10 mM HCl (μm)
Coulter Counter (*n* = 9500)	4.6 ± 0.4	3.7 ± 0.4
optical microscopy (*n* = 30)	6.4 ± 0.3	4.9 ± 0.3

### Dynamic Assessment of the Coccolithophore Calcite Content

In this section, we seek to evidence how it is possible to use the Coulter Counter to make kinetic measurements of the calcite shell dissolution. In contrast to the above-mentioned section, the particle size is not directly inferred from the size of the Coulter Counter pulses but via monitoring the time required for the shell to dissolve in a weak acid solution. Here, the calcified coccolithophores are added to a weak (acetic) acid electrolyte; this acid environment causes the carbonate shell to dissolve while the particles are in suspension. The Coulter Counter is subsequently used to monitor this dissolution process occurring in the bulk solution phase and thus enables the end point of the reaction to be determined by monitoring the measured change in the particle size. From knowledge of the end point, the initial average calcite content per cell can be independently determined. Herein, we refer to this newly developed measurement technique as the “dynamic assessment” of the particle calcite content so as to highlight that the process is a kinetic measurement and the fact that the particle size is changing over the course of the experiment.

Under acidic conditions, calcite is driven to dissolve, and this reaction is caused by both weak and strong acids. In the former case, the reaction is
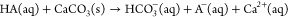
1where A^–^ and HA are the deprotonated and protonated weak acid species, respectively. For a non-adsorbing carboxylic acid such as acetic acid, the heterogeneous rate constant for this reaction is 2.5 × 10^–4^ m s^–1^.^20^ This high interfacial rate constant means that even on the micron scale, the dissolution reaction will be under near-full mass-transport control.

We seek to monitor the calcite dissolution using the Coulter Counter. To do this, a solution containing 4% NaCl, 1 mM acetic acid, and 10 mM acetate was used as the electrolyte for the Coulter Counter. Although the acetic acid is the reagent causing the calcite dissolution, the presence of the acetate plays an important role in raising the solution-phase pH to 5.4 and ensuring that the concentration of free protons is minimized. Note that the Tris buffer, Ca^2+^, and HCO_3_^–^, as discussed in the previous section, are omitted from the electrolyte as we wish to measure the dissolution of calcite “dynamically” as a function of time during the experiment. Experimentally, the Coulter Counter was set to a flow rate of ∼20 μL s^–1^ and to record every resistive pulse. Initially, no phytoplankton were present in the solution for analysis, and after approximately 10 s, an *E. huxleyi* sample was added to the analyte containing 1mM acetic acid. Upon addition of the coccolithophores to the electrolyte, their shells start to dissolve. As the reaction proceeds, the size of the particles passing through the Coulter Counter orifice decreases.

**Figure 4:**
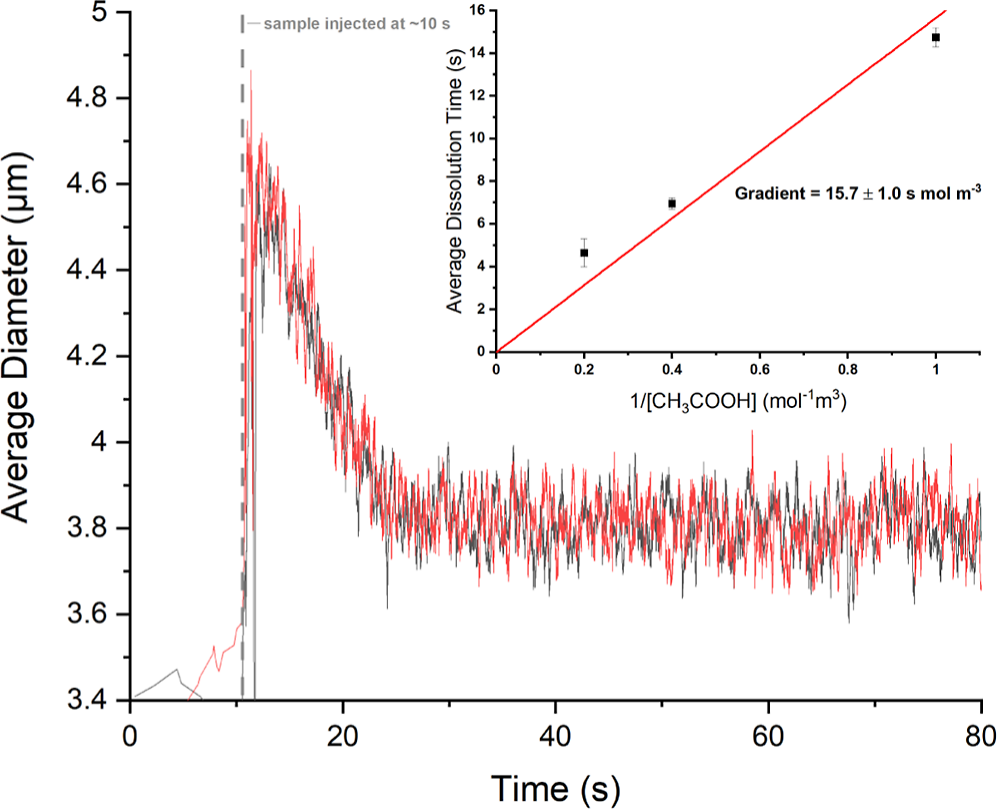
Plot of the mean particle size as a function of time as reported using the Coulter Counter for two technical replicates (red and black lines). Note the coccolithophore sample is injected into the electrolyte at ∼10 s after the onset of the measurement; this leads to the jump in the measured particle size. Due to the mild acid conditions in the electrolyte, the calcite shells of the coccolithophore shells dissolve leading to a decrease in the measured particle size. This dissolution requires 14.7 ± 0.4 s to complete. Here, 1 mM acetic acid is used to dissolve the carbonate shells of an *E. huxleyi* sample (day 6 of incubation). The inlay shows the variation of the rate with the acetic acid concentration (1/2.5/5 mM acetic acid and 10 mM acetate, where the latter is added to minimize free protons in the solution phase).

Figure 4 presents a time-windowed average of the particle size during the course of the experiment. The Experimental Section gives full details on the filtering and data analysis procedure. As can be seen in Figure 4, after addition of the phytoplankton sample, the average phytoplankton relative diameter was found to be 4.7 μm, and over the course of a few seconds, the measured average diameter decreases to 3.8 μm. These measured sizes, at the point of injection and tens of seconds after injection, represent the relative size of the phytoplankton with and without a calcite shell as measured using the Coulter Counter in the static mode using an excess of a strong acid (cf. Figure 3 and above). Furthermore, the measured diameter decreases essentially linearly with time until the final decalcified size is reached. This measurement is highly reproducible where Figure 4 shows the overlay of two technical replicates (red and black lines). From this data, it was determined that dissolution of the calcite shell by the acetic acid solution required 14.7 ± 0.4 s.

Assuming that the dissolution rate, *J*_Dis_ (mol s^–1^), is first-order with respect to the acid concentration
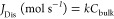
2where *k* (m^3^ s^–1^) is some, as of yet, unknown rate constant and *C*_bulk_ is the bulk concentration of acetic acid, such that
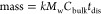
3where *t*_dis_ is the time taken for the reaction to occur, *M*_w_ is the molecular weight of the solid (100.1 g mol^–1^ for calcite), and mass is the calcite mass on a cell. On rearranging eq 3, we get
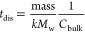
4

From this analysis, it can be seen that the dissolution time should be inversely proportional to the acid concentration. The inlay of Figure 4 presents the average dissolution time as a function of the inverse of the acetic acid concentration (see Supporting Information Section S4 for representative examples of the raw experimental data), hence evidencing that the dissolution rate is proportional to the acetic acid concentration. The gradient of this plot has a value of 15.7 ± 1 mol m^–3^ s, and in accordance with eq 4, this corresponds to the constant mass/*kM*_w_. Notably, there is some indication in data presented in the inlay of Figure 4 that at higher acetic acid concentrations, the dissolution reaction occurs at a slower rate than may be expected on the basis of eq 4; this is reflected in the non-zero intercept of the experimental data. Mechanistically, this may indicate that at higher acid concentrations, the reaction rate approaches the measurable limit of the Coulter technique, plausibly leading to an artificial overestimation of the calcite dissolution time. Note that in the absence of an acetic acid buffer, the calcite shell dissolution reaction takes approximately an order of magnitude longer, such that over the course of ∼20 s, there is only minimal (∼10%) dissolution of the shell (see Supporting Information Section S5 for full details).

Given the high reproducibility of this in situ acid-driven kinetic measurement (see Figure 4 for the overlay of two technical replicates of the measurement), to what extent can the dissolution time be used to quantify the average cellular calcite content? First, the mass-transport-limited flux density of a spherical particle increases inversely with respect to the particle size.^21,22^ For small (micron-sized) particles, the diffusional mass transport can be viewed as being at a steady-state limit. Furthermore, for the case in which the kinetics of the dissolution process is finite, the interfacial reaction rate needs to be accounted for. Consequently, in the situation where the dissolution reaction (eq 1) is under a mixed kinetic regime,^23^ both the interfacial reaction kinetics and the mass transport of the acetic acid contribute to the overall reaction rate. Then, taking a diffusion-only mass-transport model, the dissolution rate, *J*_Dis_ (mol s^–1^), for this acetic acid-driven dissolution reaction is defined analytically as
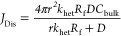
5where *r* is the particle radius (m), *k*_het_ is the heterogeneous rate constant (m s^–1^) for the reaction, *R*_f_ is the roughness factor, a measure of the surface area of the calcite-encrusted surface relative to that of an equivalently sized sphere, and *D* is the diffusion coefficient of acetic acid (m^2^ s^–1^) (see Supporting Information Section S6 for a full derivation). In the above-mentioned analysis, the roughness factor is an important quantity effectively modulating the interfacial reaction rate. The heterogeneous rate constant for the dissolution process has been reported to be 2.5 × 10^–4^ m s^–1^; however, this rate has been measured at a flat surface, and it must be recognized that the calcite on the coccolithophores is nanostructured. If the calcite shell surrounding the coccolithophore was a smooth sphere, then the roughness factor would have a value of 1; however, in the present case, the calcite surface that is accessible during the dissolution reaction is clearly greater than that of a smooth sphere, and hence, *R*_f_ must have a value greater than unity. A roughness factor of 4 ± 2^24^ has been previously advocated for in the literature on the basis of optical dissolution study experiments; hence, in the following analysis, we use this range of roughness factors. Furthermore, it should be commented that eq 5 varies as a function of the particle radius, and it is important to recognize that this radius refers to the geometric particle size as can be measured accurately by optical microscope and not the relative particle size as reported using the Coulter Counter. Furthermore, as the particle shell is dissolved, the particle radius will decrease reducing the rate of dissolution. In the following analysis, we assume that the particle radius is well described by that measured optically and varies linearly as a function of time during the dissolution process as seen in Figure 4. Importantly, from eq 5, it can be shown that if the dissolution reaction is controlled by the interfacial kinetics of the calcite dissolution processes, the particle diameter is theoretically expected to decrease linearly as a function of time. On the basis of these assumptions, the use of the measured dissolution time (14.7 ± 0.4 s for the present example shown in Figure 4), as experimentally measured via the Coulter Counter, and integration of eq 5 yield a measure of the calcite mass per cell (see Supporting Information Section S6 for further details). For example, for the data presented in Figure 4, a mass of 39.2 ± 8.1 pg per cell was calculated for the *E. huxleyi* sample on day 6 of incubation. Here, the confidence interval reflects the inaccuracy in the roughness factor of the coccosphere shell. Given that this roughness factor will have a true value which we can only estimate, the inaccuracy in this parameter represents a significant additional variance in the calculated calcite mass. This inaccuracy in the roughness factor is significantly larger than the uncertainty in the dissolution time as determined from the Coulter Counter measurement. Notably, this kinetic methodology for determining the calcite mass is not restricted to the analysis of the soft calcified particles. Successful adaption of the technique for determining the mass of other materials will, in part, be dependent on ensuring the stability of the particle suspension in the high-ionic strength electrolyte; the derivation of eq 5 assumes the particles to be diffusionally isolated and independent. Having outlined the analytical procedure for analyzing the dynamic coccolithophore data, this work now turns to consider the validation of this measurement and the assessment of the calcite coccolithophore mass for a range of different samples.

### Validation of the Coulter Counter Kinetic Measurement

**Figure 5:**
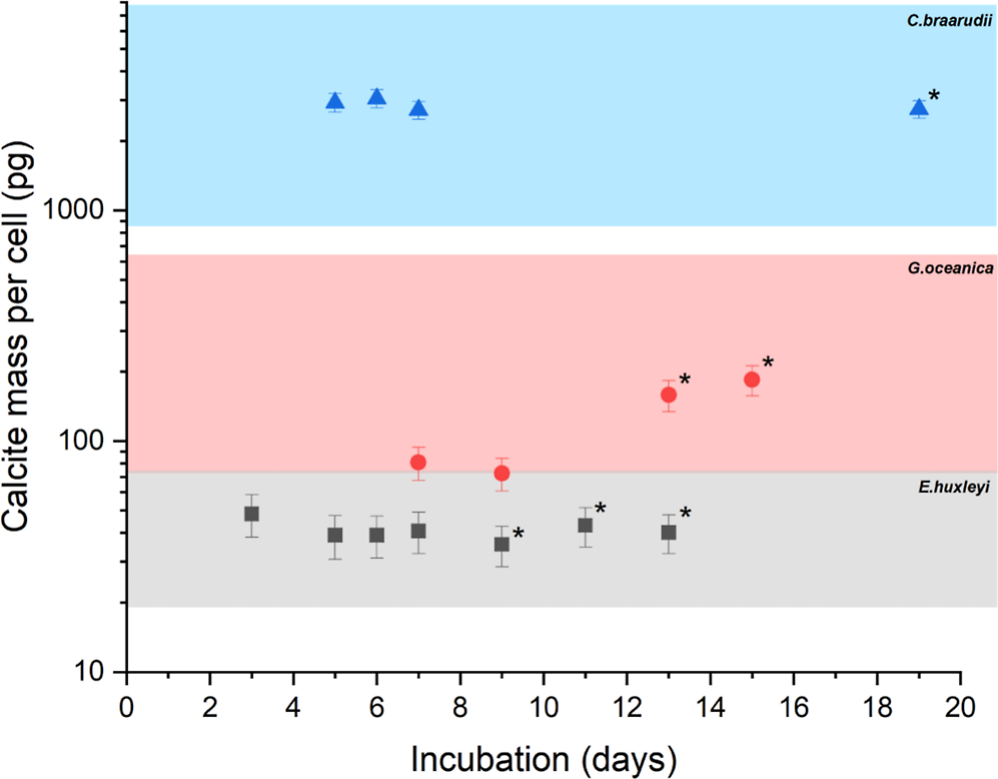
Calcite mass per cell as referred from the kinetics of the acetic acid-driven dissolution of the coccolithophore shell for the three species *E. huxleyi* (black), *G. oceanica* (red), and *C. braarudii* (blue). Also shown for direct comparison is the expected range of calcite masses as reported in the literature (colored bands). Measurements with a * indicate that the sample is in the stationary phase of its growth curve. The errors represent the inaccuracy in the measurement associated with the uncertainty in the experimental roughness factor.

Using the newly developed dynamic analytical procedure introduced above, the calcite masses of three different species, *E. huxleyi*, *G. oceanica,* and *C. braarudii*, were measured as a function of growth following the initial inoculation. Supporting Information Section S7 presents the cell counts for these three species as a function of time demonstrating that the *E. huxleyi* sample reaches the stationary phase after 7 day growth, whereas the *G. oceanica* and *C. braarudii* require approximately 12 and 15 day growth, respectively, before their growth rate reaches a plateau. Figure 5 presents the measured coccolithophore calcite masses for these three species as a function of their growth time. A roughness factor of 4 ± 2 is applied for all three species.^24^ Also overlaid is a comparison of the expected calcite mass per cell on the basis of the reported literature values (see Supporting Information Section S8). The newly presented technique is in excellent agreement with the range of values as reported in the literature for each species, and this remains to be the case despite encompassing any variation in calcite mass per cell which might be expected at different stages of the growth curve.^24,25^ As can be seen from Figure 5, the mass of calcite per cell differs by almost 2 orders of magnitude between the *E. huxleyi* and *C. braarudii* samples. Given the far greater mass of calcite associated with the *C. braarudii* sample, the experimental dissolution times were significantly longer, taking an average of 272 ± 24 s for the material to dissolve. Supporting Information Section S9 presents representative examples of the raw dissolution kinetic measurements made using the Coulter Counter for both *G. oceanica* and *C. braarudii*.

**Figure 6:**
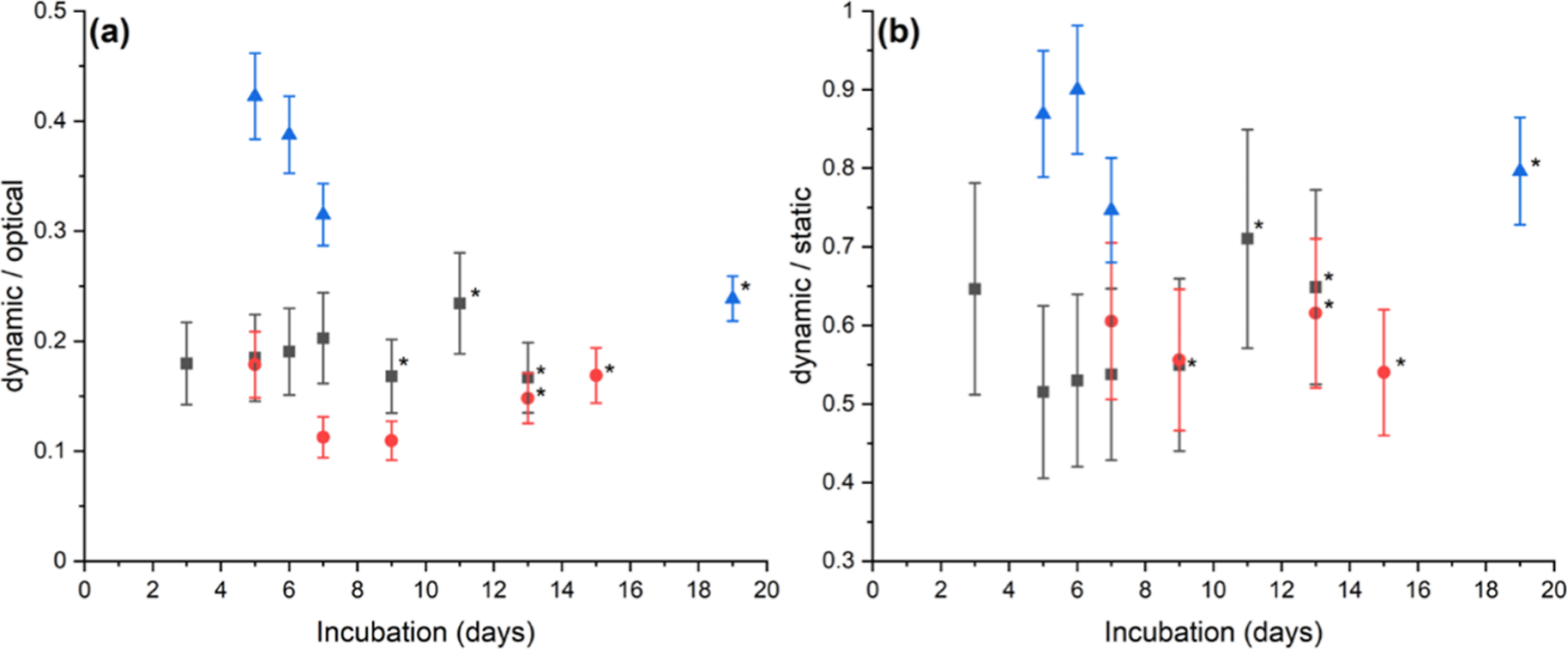
Comparison of the volumes as measured via the acetic acid (dynamic) procedure to those estimated from optical and static Coulter Counter measurements. Data is presented as the ratio of the dynamic measurement divided by the volume of either the optical (a) or static Coulter Counter (b) *E. huxleyi* (black), *G. oceanica* (red), and *C. braarudii* (blue), where the error bars represent the systematic uncertainty in the dynamic acetic acid measurement arising from the uncertainty in the experimental roughness factor. Data demarked with a * indicates measurements that have been made with a sample in the stationary phase of its growth curve. Note that the apparently larger error bars in (b) reflect the scaling of the data where the relative uncertainty in the two measurements is the same arising from the systematic error in the dynamically determined calcite cellular content.

The above demonstrates that the Coulter Counter in combination with the optical measurement of the cell size can provide a reproducible measurement of the calcite mass per cell for different coccolithophore species with calcite mass spanning 2 orders of magnitude. This kinetic measurement is achieved by using the Coulter Counter to monitor the course of the reaction and using the optical measurements of the cell size as inputs into the analytical model to enable the calcite mass to be calculated from the total reaction time. Notably, although, the Coulter Counter is used to monitor the course of the reaction, the results do not depend on the absolute magnitude of the Coulter Counter pulses but only use the change in the response to indicate when the reaction has been driven to completion. Subsequently, this work now turns to consider the relationship among the direct optical measurement of the cell volume as calculated from the particle projected area, the relative calcite volumes as reported using the Coulter Counter (static assessment), and the values determined via acetic acid dissolution (dynamic assessment). First, from the optical measurement of the cell dimensions with and without a calcite shell, it is possible to estimate the total volume of the shell. This optically determined shell volume represents the entire envelope volume of the shell corresponding to both the calcite itself and the electrolyte occluded in the structure. As a note of caution, it should be recognized that the accuracy of this optical measurement assumes that the coccolithophore cell size is not significantly altered when exposed to the mild-acid conditions. Figure 6a compares this optically measured volume to that determined for the same sample using the dynamic assessment procedure outlined above. The difference between the two measurements is given as the ratio of the shell volume measured by the acetic acid procedure divided by that estimated from the optical measurements.

As seen from the optical data, Figure 6a shows that on the basis of the calcite mass as measured via the acetic acid procedure for both *E. huxleyi* and *G. oceanica*, about 10–20% of the total volume of the shell is composed of calcite, and the remaining material will be the predominantly occluded electrolyte. In contrast, the *C. braarudii* shell is denser, and for the cells measured during their exponential growth phase, about 30–40% of the volume occluded by the shell consists of calcite. Relatedly, Figure 6b presents the ratio of the calcite volume as measured via the acetic acid procedure (dynamic assessment) divided by the volume as estimated from the differences in the cell volume measured before and after acidification (static assessment) of the measurement electrolyte. Interestingly, in all cases, the discrepancy between the two measurements is less; for both the *E. huxleyi* and *G. oceanica* samples, 50–70% of the volume reported using the Coulter Counter is calcite. Furthermore, for *C. braarudii,* the statically measured volume comprises 70–90% calcite.

It is interesting to reflect that, as highlighted in Table 1, the static Coulter Counter measurement systematically underestimates the *cellular* volume. The reasons for this underestimation were discussed above and most likely reflect the deformability of the cells as it accelerates toward the orifice.^4,19^ In contrast, as can be seen from the data presented in Figure 6b, the static Coulter Counter measurement of the *shell* volume is overestimated as compared to that of the true calcite volume. In the case of the calcite shell, the porous structure leads to the occlusion of the electrolyte in the shell, leading to an overestimation of the volume change as measured using the Coulter Counter. This overestimation of the volume of porous structures using the Coulter technique is well documented.^15,16^ The fact that the *C. braarudii* measurement is overestimated to a lesser extent, as evidenced in Figure 6b, is consistent with the data presented in Figure 6a which also indicates that the *C. braarudii* shell is denser. From the data presented in Figure 6b, we can see that a reasonably accurate estimation of the shell calcite volume can be made by making a static Coulter Counter volume measurement and then correcting the measured volume to account for the overestimation due to the occlusion of the electrolyte in the biogenic structure. The ratios presented in Figure 6b quantify this overestimation by the static Coulter Counter measurement. Note that in the Coulter Counter literature, this ratio of the overestimation of the material is referred to as a “Shape Factor”. Importantly, across the days of culture growth, we see a similarity in the shape factor between *E. huxleyi* and *G.oceanica* but not with *C. braarudii*. This indicates that while a “universal” shape factor might be plausible when applied to *E. huxleyi* and *G. oceanica*, further derivation of “shape factors” is likely to be required for other coccolithophore species. In addition, the cultures in these experiments were conducted on single strains of each species. In the case of *E. huxleyi*, for example, there are an array of different morphotypes which reflect differences in the coccolith structure and the likely per cell quota of calcite.^26,27^ Repeating the measurements presented here on a representative group of the different *E. huxleyi* morphotypes could help determine whether a “universal shape factor” is a favorable approach when applying this method to *E. huxleyi* specifically.

Obtaining rapid and reproducible measurements of per cell particulate inorganic carbon (PIC), as presented here by the measured per cell calcite mass, is of great advantage to researchers interested in quantifying cellular calcification and its variability in culture experiments. This is of particular relevance to those investigating the effects of various environmental perturbations on cellular carbon allocation, as fluctuations in environmental conditions are likely to drive changes in the morphology and extent of calcification.^10,25,28^ Current widely used methods to obtain PIC often require the time-consuming and costly process of preparing samples for elemental analysis,^10,18^ where acid treatment of duplicated samples is necessary to determine total inorganic carbon from total particulate carbon with inherent propagation of errors from the ratioing of two independent representative samples. The bulk method proposed here, while limited to having access to a Coulter Counter, offers a novel solution to obtain rapid estimates of coccolithophore PIC concurrently with a cell count for monitoring a culture. This would allow additional insights into the variation of calcification during growth under a range of conditions, in addition to providing cell size. The high-throughput nature of this approach could greatly cut time and laboratory costs, serving as great advancement for those working with an array of experimental treatments and with a high number of replicated cultures.

In the future, however, it will be necessary to assess if there is significant variability of the shape factor for the same species under different environmental conditions to validate that the methodology can capture changes in calcification which are independent of the shape factor. It will be necessary to assess which dimensions of calcification control the shape factor (e.g., thickness of calcite elements) versus the volume occluded by the shell (e.g., number of liths per cell). In order to understand how the environment influences physiology and could trigger a change in total population calcite production rates,^28,29^ it will be the key to assess which parameters drive variable calcite per cell by the “intensity of calcification” of the liths or the number of liths per cell via the growth rate. These links may also provide additional insights into the physiological mechanisms driving changes in calcification. Nonetheless, the ability to measure the cell number, cell volume, and calcite per cell concurrently will transform our ability to gain real-time insights into calcification production rates during population growth of coccolithophores in response to environmental change.

## Conclusions

Coulter Counters provide a rapid method by which coccolithophore cells can be counted, but they also provide a relative measure of cell volume. This volume measurement is sensitive to the presence of the calcite shell such that the dissolution of the biogenic material can be monitored using the technique. In a 1 mM acetic acid solution, the calcite shell surrounding a coccolithophore is dissolved over the course of tens to hundreds of seconds depending on the size and calcite content of the cells in a sample. This dissolution of the calcite shell occurs in the particle suspension prior to the material traversing the Coulter Counter’s orifice, such that the Coulter Counter is solely used to determine the time required for the dissolution to run to completion. Hence, this new methodology enables a kinetic measurement of the average calcite mass per cell, and importantly, the determined value is not subjected to the errors arising from distortion of the cell or occlusion of the electrolyte in the calcite structure as commonly occurs with a conventional static assessment of the calcite volume. Herein, using this new methodology, we monitor the average calcite mass per cell of three different coccolithophore species during their growth in a laboratory environment wherein we demonstrate that the technique is capable of measuring calcite masses that span almost 2 orders of magnitude where for *E. huxleyi*, *G. oceanica*, and *C. braarudii,* the average masses of 41, 120, and 2900 pg per cell are measured, respectively. This in situ measurement allows us for the first time to provide a *direct* route by which the relative shell volume, as measured from the Coulter Counter pulse heights, can be calibrated and corrected. This correction factor will account for a number of effects including overestimation of the shell volume due to the occlusion of the electrolyte and possible changes in the measured cell volume due to possible changes in the cell deformability before and after dissolution of the calcite material. Importantly, we also show that the required correction factor is similar for *E. huxleyi* and *G. oceanica*, where shape factors of 0.59 and 0.58 are found, respectively; however, for *C. braarudii,* a shape factor of 0.83 is determined. In the latter case, this higher shape factor likely reflects the denser structure associated with the liths encrusting the cells.

## Supporting Information Available

Summary of the literature view on Coulter Counter sensitivity to biogenic calcite, experimental details on coccolithophore culturing conditions, evidence that the acid created using the Coulter Counter can interfere with the measurement, control experiments performed in the absence of acetic acid and with variable acetic acid concentrations, derivation of the mixed kinetic regime model, experimental growth curves for the studied plankton, literature values for the mass of calcite per cell for the three species, and additional experimental data for two of the coccolithophores (PDF)
