# Supplementary material for: Quantifying the Extent of Calcification of a Coccolithophore Using a Coulter Counter
Source: Anal Chem. 2022 Sep 8;94(37):12664–72. doi: 10.1021/acs.analchem.2c01971 (PMC9494302; doi:10.1021/acs.analchem.2c01971)
Supplement: Supplementary file 1 — ac2c01971_si_001.pdf [file ac2c01971_si_001.pdf]

Supporting Information:

Quantifying the Extent of Calcification of a Coccolithophore Using a Coulter Counter

Xinmeng Fan[1], Christopher Batchelor-McAuley[1], Minjun Yang[1], Samuel Barton[2], Rosalind E.M. Rickaby[2], Heather A. Bouman[2], Richard G. Compton[1*]

[1] Physical and Theoretical Chemistry Laboratory, Department of Chemistry, University of Oxford, South Parks Road, Oxford OX1 3QZ, Great Britain.

[2] Department of Earth Sciences, University of Oxford, South Parks Road, Oxford OX1 3AN, Great Britain.

* corresponding author email: Richard.Compton@chem.ox.ac.uk

Table of Contents

Section 1: Literature views about coccolithophores characterisation measured by a Coulter Counter 2

Section 2: Coccolithophore culturing conditions 4

Section 3: Acid generated by the Coulter Counter dissolved calcite shell in the absence of a buffer..6

Section 4: Dissolution of the calcite shell in differing acetic acid concentrations 8

Section 5: Dissolution of the calcite shell in the absence of an acetic acid buffer 9

Section 6: Derivation of the dissolution rate, J_{Dis} (mol s^{-1}) and calcite mass per cell 11

Section 7: Growth curves of three different plankton species 13

Section 8: Expected range of calcite masses reported in the literature 14

Section 9: Experimental dissolution kinetic for *G.oceanica* and *C.braarudii* 15

Section 1: Literature views about coccolithophores characterisation measured by a Coulter Counter

Table S1: A summary of the literature views about the Coulter Counter.

Work Done By	Able to Measure	Not Able to Measure
Iglesias-Rodriguez et al., 2008[1]	Coccolith volume, Coccosphere volume	-
Oviedo et al., 2014[2]	Cell density, Cell volume	Coccosphere volume
Aloisi, 2015[3]	Cell density, Cell volume	Coccosphere volume
McClelland et al., 2016[4]	Coccolithophore volume and concentration	
Bretherton et al., 2019[5]	Coccosphere diameter, Cell diameter	-
Müller et al., 2008[6], 2015[7] and 2021[8]	Cell number, Coccolith volume, Coccosphere volume, Cell volume	-

Table S1 summarises recent views about coccolithophores characterisation as measured by a Coulter Counter. Some work has reported that the coccosphere volume as measured by the Coulter Counter is smaller than that obtained from other techniques and they provide differing explanations for these apparent inconsistencies:

- Iglesias-Rodriguez et al., 2008[1] noted that the coccosphere volume measured by the Coulter Counter is smaller than the one measured by the flow cytometry. They explain that this may be due to the different analytical procedures used in the techniques.
- Oviedo et al., 2014[2] and Aloisi, 2015[3] also reported that the measured coccosphere diameter by the Coulter Counter is smaller than the diameter measured by the SEM images and as compared to that calculated from the geometric model respectively. They argue that the technique does not register the coccosphere because the electrolyte possibly penetrates into the layer of coccoliths and the Coulter Counter would treat the coccosphere as the background electrolyte.

Other articles in the literature have attempted to use the Coulter Counter to quantify the size and volumes of calcified coccolithophores:

- McClelland et al., 2016[4] used the Coulter Counter technique to measure the coccosphere and cell volume.

- Bretherton et al., 2019[5] utilized the Coulter Counter to measure the mean diameter of the coccolithophores before and after acid treatment and from this estimated the thickness of the coccosphere via the difference of the mean diameter.
- Müller et al., 2008[6], 2015[7] and 2021[8] used the Coulter Counter to measure the coccolith, coccosphere and cell volume. They believed that the small gaps between the coccoliths theoretically could be 'read' by the Coulter Counter when the gaps are filled with the electrolyte.[8] They translated the coccolith volume to the calcite mass and that was in good agreement with the reported values.

Section 2: Coccolithophore culturing conditions

All three species of coccolithophore were grown on Aquil synthetic ocean water[9] based K/2 culture medium, with F/2 vitamin enrichment[10](See Table S2). All cultures were maintained by regularly sub-culturing into fresh growth medium under sterile conditions, and during the exponential phase of growth. Cultures were grown in a PHCbi MLR-352-PE Incubator (PHC Europe B.V.) under a 14:10 hour light-dark cycle, with PAR intensity of 20-40 µmol m^{-2} s^{-1}, and at a temperature of 17°C.

Table S2: The molar concentration of all the components in the Aquil synthetic seawater, K/2 growth medium and F/2 vitamin enrichment.

		Molar Concentration in final growth medium (mol dm^{-3})
Aquil Synthetic Ocean Water (SOW)[9]	NaCl	4.20 x 10^{-1}
	Na_2SO_4	2.88 x 10^{-2}
	KCl	9.39 x 10^{-3}
	$NaHCO_3$	2.38 x 10^{-3}
	KBr	8.40 x 10^{-4}
	H_3BO_3	4.85 x 10^{-5}
	NaF	7.15 x 10^{-5}
	$MgCl_2.6H_2O$	5.46 x 10^{-2}
	$CaCl_2.2H_2O$	1.05 x 10^{-2}
	$SrCl_2.6H_2O$	6.38 x 10^{-5}
K/2 medium enrichment based on the K recipe, silica included[10]	$NaNO_3$	4.41 x 10^{-4}
	NH_4Cl	2.50 x 10^{-5}
	Na_2 b-glycerophosphate	5.00 x 10^{-6}
	H_2SeO_3	5.00 x 10^{-9}
	$FeCl_3 \cdot 6H_2O$	5.85 x 10^{-6}
	$Na_2EDTA \cdot 2H_2O$	5.55x 10^{-5}
	$CuSO_4 \cdot 5H_2O$	5.00 x 10^{-9}
	$Na_2MoO_4 \cdot 2H_2O$	1.30 x 10^{-8}

	$ZnSO_4 \cdot 7H_2O$	4.00×10^{-8}
	$CoCl_2 \cdot 6H_2O$	2.10×10^{-8}
	$MnCl_2 \cdot 4H_2O$	4.50×10^{-7}
	$Na_2SiO_3.9H_2O$	2.52×10^{-4}
F/2 vitamin enrichment[10]	Thiamine. HCl (Vitamin B1)	2.96×10^{-7}
	Biotin (Vitamin H)	2.05×10^{-9}
	Cyanocobalamin (VitaminB12)	3.69×10^{-10}

Section 3: Acid generated by the Coulter Counter dissolved calcite shell in the absence of a buffer

In a Coulter Counter experiment a potential is applied across the two platinum foils, consequently electrolysis occurs. In the present case protons are generated at the anode (Eq1), the anode is in the coccolithophore sample and this produced acid is able to dissolve the biogenic calcite (Eq2).

$$2H_2O(l) \rightarrow O_2\,(g) + 4H^+(aq) + 4e^- \tag{1}$$

$$CaCO3\,(s) + H^+(aq) \rightarrow Ca^{2+}(aq) + HCO_3{}^-(aq) \tag{2}$$

For example, for a measurement using an 800 µA current, after one minute of measurement up to 5.0 × 10^{-7} mol of protons *may* be produced. The quantity of protons formed will depend on the efficiency of the electrode process, where in the presence of chloride the electro-generation of chlorine will be a competing process. However, if initially we assume 100% efficiency and taking an example Coulter Counter cell volume of 20 mL, then every minute the proton concentration may increase by up to 25 µM.

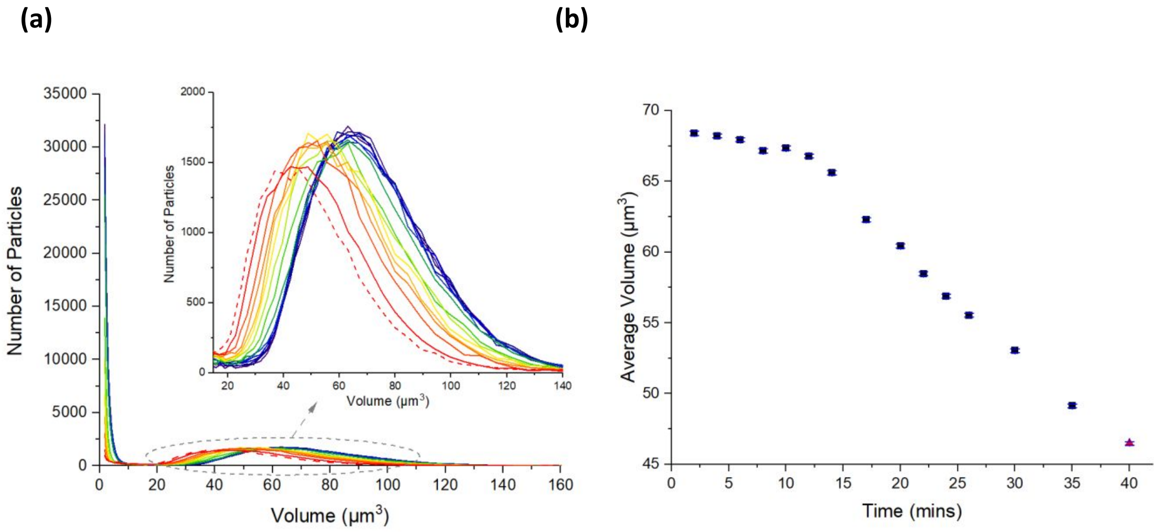

Figure S1: Size distribution of E.huxleyi measured by the Coulter Counter. Solid lines in (a) are the measured distribution from 2mins after adding the sample to 35mins (from blue to red). Red dash line in (a) is in the presence of an additional 5mM HCl to the electrolyte. For each measurement, average volume of the coccolithophores is calculated and plotted as a function of time in (b). Blue bars represent the standard deviations and red triangle at 40mins is in the presence of an additional 5mM HCl.

To experimentally investigate the potential for this adventitiously produced acid to react with the biogenic calcite, a solution containing 4% NaCl, 20 mM $CaCl_2$ and 1 mM $NaHCO_3$ adjusted to pH 8 was used as the electrolyte in the Coulter Counter. 0.5mL of the *E.huxleyi* sample was added to 20mL of the electrolyte.

The size distribution of the coccolithophore sample was measured using the Coulter Counter 15 times in 40mins. For each run, 1mL of the sample was taken for the analysis and the measurement took ~1min to complete. As shown in Figure S1 the measured volume of coccolithophores decreased as the number of experiments progressed.

If either a larger electrolyte volume is used or if the Coulter Counter cell contains 10 mM Tris buffer then this effect of calcite dissolution can be avoided, which is shown in Figure S2.

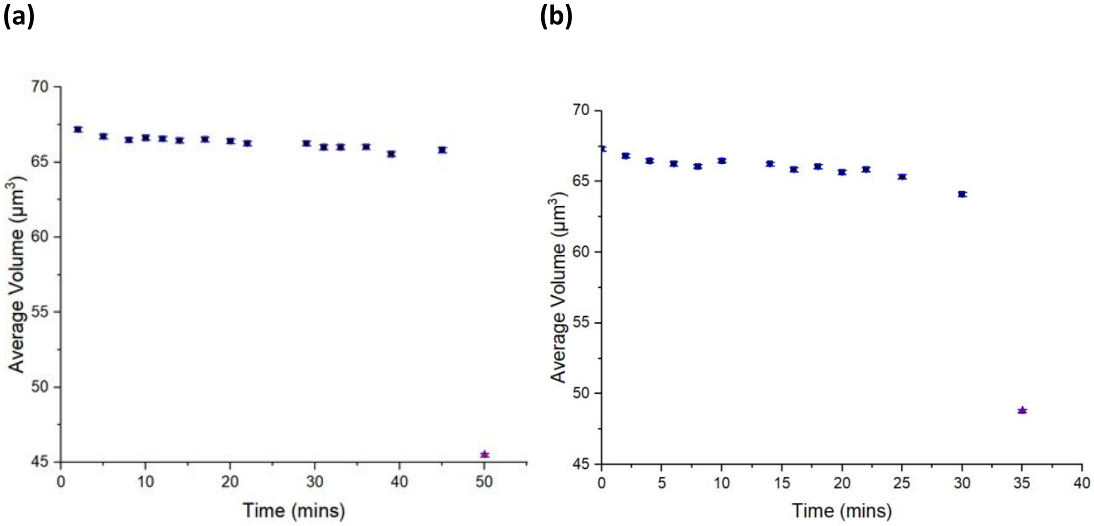

Figure S2: Average volume of E.huxleyi measured by the Coulter Counter in (a)100mL 4% NaCl, 20 mM $CaCl_2$ and 1 mM $NaHCO_3$ adjusted to pH 8 and (b)20mL 4% NaCl, 10mM TRIS buffer, 20 mM $CaCl_2$ and 1 mM $NaHCO_3$ adjusted to pH 8. Blue bars represent the standard deviations and red triangle is in the presence of an additional 5mM HCl.

Section 4: Dissolution of the calcite shell in differing acetic acid concentrations

Figure S3 shown below depicts representative dissolution kinetic measurements for an E.huxleyi sample as a function of acetic acid concentration (1-5 mM) .

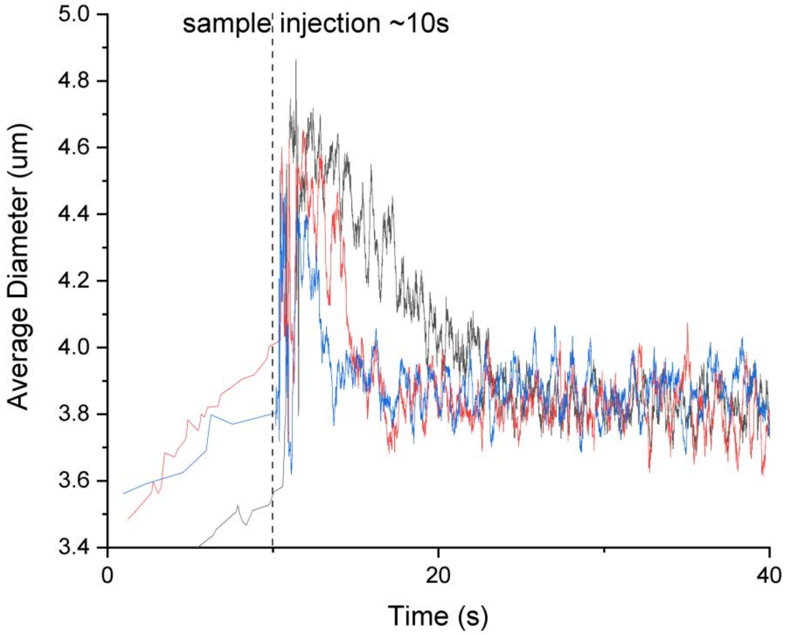

Figure S3: Acetic acid dissolution of the E.huxleyi sample on Day 6 as a function of the acetic acid concentration; black 1 mM, red 2.5 mM and blue 5 mM. Acetate concentration has been kept constant at 10 mM.

Section 5: Dissolution of the calcite shell in the absence of an acetic acid buffer

First the size of the coccolithophore was assessed using the static technique (See details in static assessment in the main text, where the coccolithophore size distribution in the absence and presence of 10mM HCl) so as to provide a measurement of the size distribution of the *E.huxleyi* sample, with and without a shell. These static Coulter Counter size distributions are shown in Figure S4. Here we use an electrolyte of 4%NaCl, 10 mM Tris Buffer, 20 mM $CaCl_2$ and 1.0 mM $NaHCO_3$ where the electrolyte has been adjusted to pH 8.0. In the absence of the acid, a relative diameter of 5.1±0.4 µm was measured and in the presence of 10 mM HCl, the calcite shell dissolved and the diameter dropped to 4.0±0.4 µm.

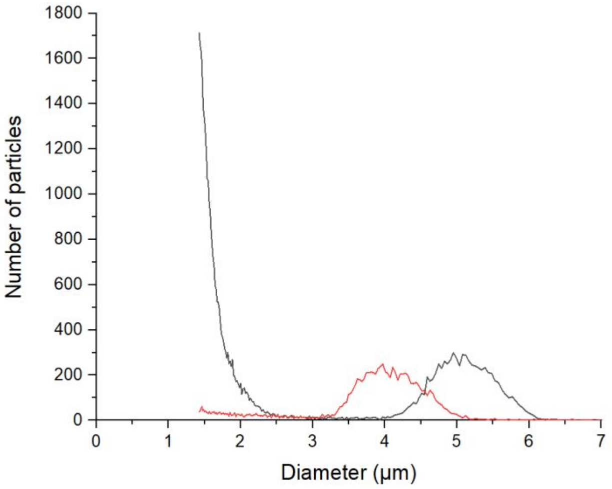

Figure S4: Size distribution of the E.huxleyi sample. Black line is in the absence of acid and red line is the presence of acid.

On the same sample, we subsequently performed a dynamic Coulter Counter measurement but in the absence of acetic acid buffer. (See details in dynamic assessment in the main text – filter the raw pulses and plot windowed averages of the particle size with time) Figure S5 shows the first 20s of the dynamic particle measurements after sample injected to the electrolyte. Over this timeframe only ~10% of the calcite shell has dissolved. Here the dissolution is driven by the undersaturation of the solution with respect to calcite.

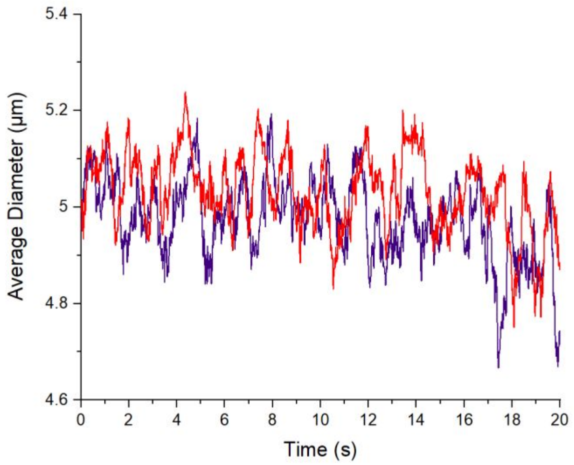

Figure S5: First 20s of the calcite shell dissolution in 4% NaCl as measured by the Coulter Counter.

Section 6: Derivation of the dissolution rate, J_{Dis} (mol s^{-1}) and calcite mass per cell

This section provides details on the derivations and calculations made in the main text.

Dissolution Rate, J_{Dis} (mol s^{-1})

Calcite dissolution requires first the acetic acid to diffuse to the surface of the coccolithophores and then the calcium carbonate to dissolve due to acid reaction:

$$[CH_3COOH]_{bulk} \rightleftarrows [CH_3COOH]_{surface} \tag{3}$$

$$[CH_3COOH]_{surface} + CaCO3 \xrightarrow{k} [Ca^{2+}]_{surface} + [HCO3^-]_{surface} + [CH_3COO^-]_{surface} \tag{4}$$

where k is the heterogeneous rate constant (m s^{-1}) for the first-order interfacial reaction.

The dissolution reaction is under a mixed kinetics regime, where the mass-transport of the acetic acid and the interfacial reaction both contributes to the overall rate. Natural convection and migration are assumed to be negligible so we only consider diffusion for this case. The total flux for each process is:

$$J_{Diffusion} = 4\pi D[C_{bulk} - C_{surf}]r \tag{5}$$

$$J_{Reaction} = 4\pi r^2 k C_{surf} R_f \tag{6}$$

where D is the diffusion coefficient of acetic acid (m^2 s^{-1}), C_{bulk} is the bulk concentration of acetic acid, $C_{surface}$ is the surface concentration of acetic acid, r is the particle radius (m) and R_f is the roughness factor - a measure of the surface area of the calcite encrusted surface relative to that of an equivalently sized sphere. In equation 6 we have assumed that the interfacial reacton kinetics for the dissolution follow first order kinetics, this is consistent with the experimental data provide in Figure 4 of the main text and further mirrors work previous reported in the literature.[11] Further, in both equations 5 and 6 we only consider the reaction occur due to direct reaction with the acetic acid. In this work this is reasonable. Experimentally we use a solution that contains 1 mM acetic acid and 10 mM acetate, the acetate serves an important role in increasing the pH of the solution of pH 5.4 and hence minimising the bulk solution phase concentration of free protons. Although protons diffuse almost an order of magnitude faster than acetic acid (D_{H+} = ~5x10^{-9} m^2 s^{-1} under aqueous high salt conditions) the proton concentration is over two orders of magnitude less than that of acetic acid.

Using steady-state approximation, if we set

$$\frac{d[CH_3COOH]_{surf}}{dt} = 4\pi D[C_{bulk} - C_{surf}]r - 4\pi r^2 k C_{surf} R_f = 0 \tag{7}$$

Then,

$$C_{surf} = \frac{DC_{bulk}}{rkR_f + D} \tag{8}$$

Substituting Eq8 into Eq6, we get

$$J_{Dissolution} = \frac{4\pi r^2 k R_f D C_{bulk}}{r k R_f + D} \ (mol\, s^{-1}) \tag{9}$$

Calcite Mass per cell

If the radius r varies linearly with time then,

$$Mass = -Mw \times \int_{t_{initial}}^{t_{final}} J_{Dis}(r(t))\, dt \approx Mw \times \frac{J_{Dis}(r(t_{inital})) - J_{Dis}(r(t_{final}))}{2} \times t \tag{10}$$

For example, for *E.huxleyi* sample on day6 of incubation with roughness factor R_f=4,

$$Mass \approx Mw \times \frac{J_{Dis}(r(t_{inital})) - J_{Dis}(r(t_{final}))}{2} \times t = 100.09 g\, mol^{-1} \times \frac{1}{2}\left[\frac{4\pi \times (3.21 \times 10^{-6} m)^2 \times 2.50 \times 10^{-4} ms^{-1} \times 4 \times 1.01 \times 10^{-9} m^2 s^{-1} \times 1 molm^{-3}}{3.21 \times 10^{-6} m \times 2.50 \times 10^{-4} ms^{-1} \times 4 + 1.01 \times 10^{-9} m^2 s^{-1}} - \frac{4\pi \times (2.46 \times 10^{-6} m)^2 \times 2.50 \times 10^{-4} ms^{-1} \times 4 \times 1.01 \times 10^{-9} m^2 s^{-1} \times 1 molm^{-3}}{2.46 \times 10^{-6} m \times 2.50 \times 10^{-4} ms^{-1} \times 4 + 1.01 \times 10^{-9} m^2 s^{-1}}\right] mol\, s^{-1} \times 14.75\, s = 39.2\, pg$$

where 100.09 g mol^{-1} is the molecular weight of calcite, 3.21×10^{-6} m and 2.46×10^{-6} m is the optical radius with and without calcite shells, 2.50×10^{-4} m s^{-1} is the heterogeneous rate constant for non-absorbing carboxylic acid, 1.01×10^{-9} m^2 s^{-1} is the diffusion coefficient of acetic acid, 1.0 mol m^{-3} is the bulk concentration of acetic acid.

Section 7: Growth curves of three different plankton species

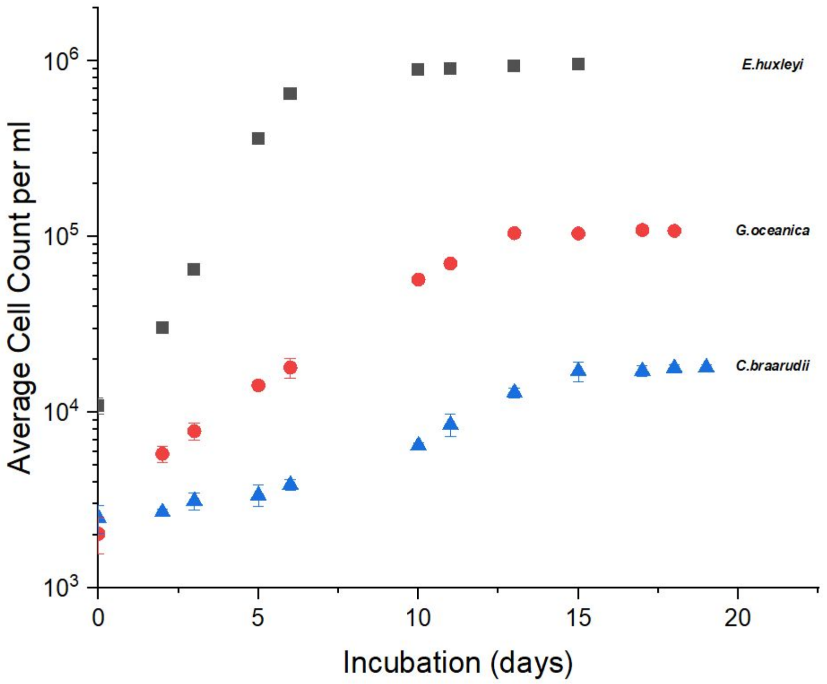

Figure S6: Growth curves of three plankton species.

For every 1-2 days, we tracked the cell count of three species via the Coulter Counter from the day of culturing to the day they reached the stationary phase. (Figure S6) For each day, 1mL of the culture is added to 9mL of the diluent (1 in 10 dilution). The diluted culture was then measured by the Coulter Counter three times. 0.1mL of the diluted culture was extracted by the Coulter Counter each time. Cell count shown on the Figure S6 represents the count for the undiluted sample, which equals to the raw cell count measured multiplying 100.

For the acetic acid dissolution experiments, the samples in exponential phase and stationary phase were studied. The *E.huxleyi* sample reaches the stationary phase on day 7, whereas the *G.oceanica* and *C.braarudii* need approximately 12 and 15 days of growth respectively before they stop growing.

Section 8: Expected range of calcite masses reported in the literature

The expected masses of calcites shells for three species is calculated by the product of the number of coccoliths and the average mass per coccoliths. The results are shown in Table S3.

Table S3: Expected masses of calcite shells based on the literature

Species	Number of Coccoliths	Average Mass per Coccolith/pg	Expected Mass of $CaCO_3$/pg
E.huxleyi	12 - 15[12]	1.5 - 5.1[13]	18.0 - 76.5
G.oceanica	10 - 24[13]	7.2 - 23.1[13]	72.0 - 554.4
C.braarudii	9 - 20[14]	99.5 - 398.6[15]	895.5 – 7972.0

Section 9: Experimental dissolution kinetic for *G.oceanica* and *C.braarudii*

Figure S7 shown below depicts representative dissolution kinetic measurements for both *G.oceanica* and *C.braarudii*. It takes 34.2±0.5s for *G.oceanica* on Day 13 and 289.0±23.4s for *C.braarudii* on Day7 to dissolve the calcite shell respectively.

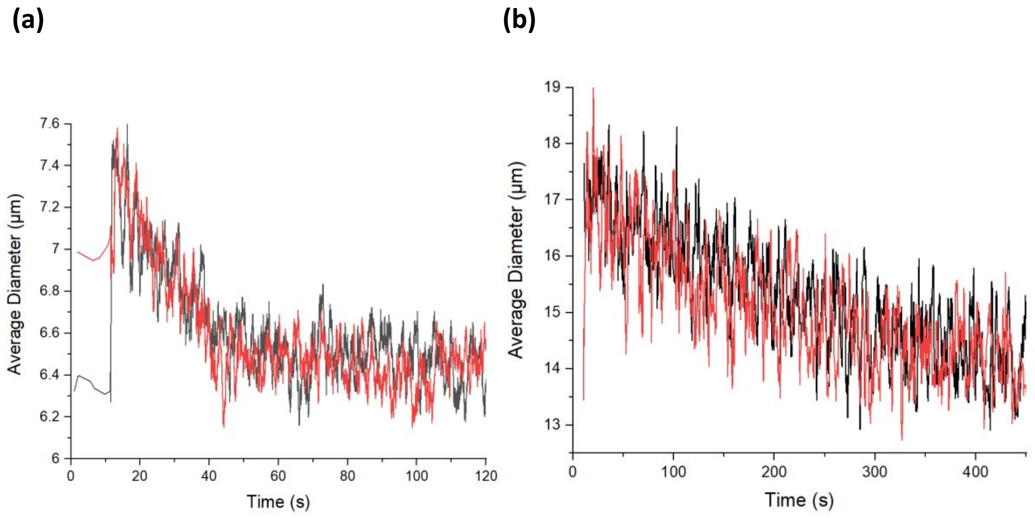

Figure S7: Acetic acid dissolution of (a) G.oceanica sample on Day 13 of the incubation and (b) C.braarudii sample on Day 7 of the incubation. Lines of different color represent repeated measurements.

References

(1) Iglesias-Rodriguez, M. D.; Halloran, P. R.; Rickaby, R. E. M.; Hall, I. R.; Colmenero-Hidalgo, E.; Gittins, J. R.; Green, D. R. H.; Tyrrell, T.; Gibbs, S. J.; Dassow, P. v.; et al. Phytoplankton Calcification in a High-CO_2 World. *Science* **2008**, *320*, 336-340.
(2) Oviedo, A. M.; Langer, G.; Ziveri, P. Effect of phosphorus limitation on coccolith morphology and element ratios in Mediterranean strains of the coccolithophore Emiliania huxleyi. *J. Exp. Mar. Bio. Ecol.* **2014**, *459*, 105-113.
(3) Aloisi, G. Covariation of metabolic rates and cell size in coccolithophores. *Biogeosciences* **2015**, *12*, 4665-4692.
(4) McClelland, H. L. O.; Barbarin, N.; Beaufort, L.; Hermoso, M.; Ferretti, P.; Greaves, M.; Rickaby, R. E. M. Calcification response of a key phytoplankton family to millennial-scale environmental change. *Sci. Rep.* **2016**, *6*, 34263.
(5) Bretherton, L.; Poulton, A. J.; Lawson, T.; Rukminasari, N.; Balestreri, C.; Schroeder, D.; Mark Moore, C.; Suggett, D. J. Day length as a key factor moderating the response of coccolithophore growth to elevated pCO2. *Limnol. Oceanogr.* **2019**, *64*, 1284-1296.
(6) Müller, M. N.; Antia, A. N.; LaRoche, J. Influence of cell cycle phase on calcification in the coccolithophore Emiliania huxleyi. *Limnol. Oceanogr.* **2008**, *53*, 506-512.
(7) Müller, M. N.; Trull, T. W.; Hallegraeff, G. M. Differing responses of three Southern Ocean Emiliania huxleyi ecotypes to changing seawater carbonate chemistry. *Mar. Ecol. Prog. Ser.* **2015**, *531*, 81-90.
(8) Müller, M. N.; Brandini, F. P.; Trull, T. W.; Hallegraeff, G. M. Coccolith volume of the Southern Ocean coccolithophore Emiliania huxleyi as a possible indicator for palaeo-cell volume. *Geobiology* **2021**, *19*, 63-74.
(9) Morel, F. M. M.; Rueter, J. G.; Anderson, D. M.; Guillard, R. R. L. Aquil: A Chemically Defined Phytoplankton Culture Medium for Trace Metal Strudies. *J. Phycol.* **1979**, *15*, 135-141.
(10) Keller, M. D.; Selvin, R. C.; Claus, W.; Guillard, R. R. L. Media for the Culture of Oceanic Ultraphytoplankton. *J. Phycol.* **1987**, *23*, 633-638.
(11) Compton, R. G.; Pritchard, K. L.; Unwin, P. R.; Grigg, G.; Silvester, P.; Lees, M.; House, W. A. The effect of carboxylic acids on the dissolution of calcite in aqueous solution. Part 1.—Maleic and fumaric acids. *J. Chem. Soc., Faraday Trans. 1* **1989**, *85*, 4335-4366.
(12) Yang, M.; Batchelor-McAuley, C.; Barton, S.; Rickaby, R. E. M.; Bouman, H. A.; Compton, R. G. Opto-Electrochemical Dissolution Reveals Coccolith Calcium Carbonate Content. *Angew. Chem. Int. Ed.* **2021**, *60*, 20999-21006.
(13) Beuvier, T.; Probert, I.; Beaufort, L.; Suchéras-Marx, B.; Chushkin, Y.; Zontone, F.; Gibaud, A. X-ray nanotomography of coccolithophores reveals that coccolith mass and segment number correlate with grid size. *Nat. Comm.* **2019**, *10*, 751.
(14) Gibbs, S. J.; Poulton, A. J.; Bown, P. R.; Daniels, C. J.; Hopkins, J.; Young, J. R.; Jones, H. L.; Thiemann, G. J.; O'Dea, S. A.; Newsam, C. Species-specific growth response of coccolithophores to Palaeocene–Eocene environmental change. *Nat. Geosci.* **2013**, *6*, 218-222.
(15) Young, J. R.; Ziveri, P. Calculation of coccolith volume and it use in calibration of carbonate flux estimates. *Deep Sea Res. Part II Top. Stud. Oceanogr.* **2000**, *47*, 1679-1700.
